# Extraction, texture analysis and polysaccharide epitope mapping data of sequential extracts of strawberry, apple, tomato and aubergine fruit parenchyma

**DOI:** 10.1016/j.dib.2018.01.013

**Published:** 2018-01-31

**Authors:** Valérie Cornuault, Sara Pose, J. Paul Knox

**Affiliations:** a Institute of Fundamental Sciences, Massey University, Palmerston North 4412, New Zealand; b Instituto de Hortofruticultura Subtropical y Mediterránea (IHSM-UMA-CSIC), Dpto. Biología Vegetal, Universidad de Málaga, 29071, Málaga, Spain; c Centre for Plant Sciences, Faculty of Biological Sciences, University of Leeds, Leeds LS2 9JT, United Kingdom

**Keywords:** Pectic polysaccharides, Fruits, Cell wall, Texture, Monoclonal antibodies, Solanaceae, Rosaceae, Tomato, Aubergine, Apple, Strawberry

## Abstract

The data included in this article are related to the research article entitled “Disentangling pectic homogalacturonan and rhamnogalacturonan-I polysaccharides: evidence for sub-populations in fruit parenchyma systems” (Cornuault et al., 2018) [1]. Cell wall properties are an important contributor to fruit texture. These datasets compile textural and immunochemical analysis of polysaccharides of four economically important fruit crops: tomato, strawberry, aubergine and apple with contrasting textures and related taxonomical origins. Cell wall components and their extractability were assessed using characterized monoclonal antibodies. In addition, textural data obtained for the four parenchyma systems show variations in the mechanical properties. The two datasets are a basis to relate cell wall composition and organization to the mechanical properties of the fruit parenchyma tissues.

**Specifications Table**

**Table:** 

Subject area	*Biology*
More specific subject area	*Plant physiology*
Type of data	*Table, Figures*
How data was acquired	*Texture analysis by texturometer through the uniaxial compression test*
	*Immunochemical data were obtained by ELISA*
Data format	*Raw data and analysis*
Experimental factors	*Portions of fruit parenchyma were cut and analyzed with a texturometer. Fruit cell wall polymers were sequentially extracted from parenchyma with water, CDTA, Na*_*2*_*CO*_*3*_*and KOH. The cell wall fractions were analyzed by ELISA to reveal partial compositions. Relationships among textural features, taxonomical origin and cell wall composition were determined.*
Experimental features	*Texturometer analysis of fruit parenchyma.*
	*Cell wall extraction yields.*
	*ELISA of sequentially extracted cell wall polysaccharides using characterised monoclonal antibodies against pectic homogalacturonan, pectic rhamnogalacturonan-1, xyloglucan and heteroxylan.*
Data source location	*University of Leeds, Leeds, UK*
Data accessibility	*Data are presented in this article*

**Value of the data**

• Data comparing cell wall composition and extractability of four fruits with high commercial values: tomato, strawberry, aubergine and apple.
• Texture analysis of tomato, strawberry, aubergine and apple parenchyma systems included Hardness (N) and Elastic modulus (kPa) and differences depending on the choice of textural parameter are compared.
• Novel data available for the cell wall composition of aubergine fruits. This dataset presents the cell wall constituents and mechanical properties of aubergine fruit parenchyma systems.

## 1 Data

### 1.1 Texture analyses and cell wall extractability of the four fruits

See Table 1: Analyses of parenchyma texture of four fruits using a texturometer. Aubergine and apple have the firmest textures, measured as the maximum force withstood by the tissues, (41 and 30 N respectively) as opposed to 7.9 and 0.7 N for tomato and strawberry. By contrast, according to the elastic modulus (*E*), apple is the stiffer tissue, followed by aubergine, with tomato and strawberry as the softer tissues. Regarding % Brix (a ripening related parameter) strawberry and apple had the highest content of soluble solids, and tomato and aubergine have a similar lower value below 4.5%. Regarding the moisture content (%), this was higher in tomato and aubergine (~93%) in contrast to the ~85% obtained in strawberry and apple. Both ripening parameters combined relate to the typical sweeter taste of strawberry and apple in contrast to tomato and aubergine that are usually considered vegetables.

**Table 1:** Analysis of four mature fruits. Texture, ripening fruit parameters and cell wall yields of four mature fruits – tomato, aubergine, strawberry and apple. Average data of 4–12 independent measurements from 3 to 5 different fruits.

**Common name**	**Family**	**Texture features**		**Ripening parameters**		**Yield**	
						**(% d.w. basis)**	
		**Hardness (*N*)**	**Elastic modulus (kPa)**	**% Brix**	**% Moisture content**	**CWM**	**Phenol-sol**
Tomato	Solanaceae	7.9 ± 1.0c	2.2 ± 0.7c	4.3 ± 0.2c	93.7 ± 0.2a	6.9 ± 0.7b	0.5 ± 0.0b
Aubergine	Solanaceae	41.1 ± 6.3a	7.4 ± 1.0b	4.5 ± 0.4c	93.4 ± 0.1a	33.8 ± 1.4a	0.3 ± 0.1b
Strawberry	Rosaceae	0.7 ± 0.1d	2.2 ± 0.3c	8.9 ± 2.0b	85.6 ± 2.3b	7.0 ± 1.1b	1.3 ± 0.2a
Apple	Rosaceae	30.3 ± 2.1b	13.5 ± 0.9a	12.0 ± 0.4a	84.9 ± 0.5b	10.3 ± 0.2b	0.2 ± 0.1b

Statistical analysis for all results was by ANOVA and Tukey-HSD, but hardness that follows a Kruskal-Wallis rank sum test and Wilcoxon rank sum test (p 0.05). CWM = cell wall material, d.w. = dry weight, phenol-sol = phenol soluble solids.

**Fig. 1:**
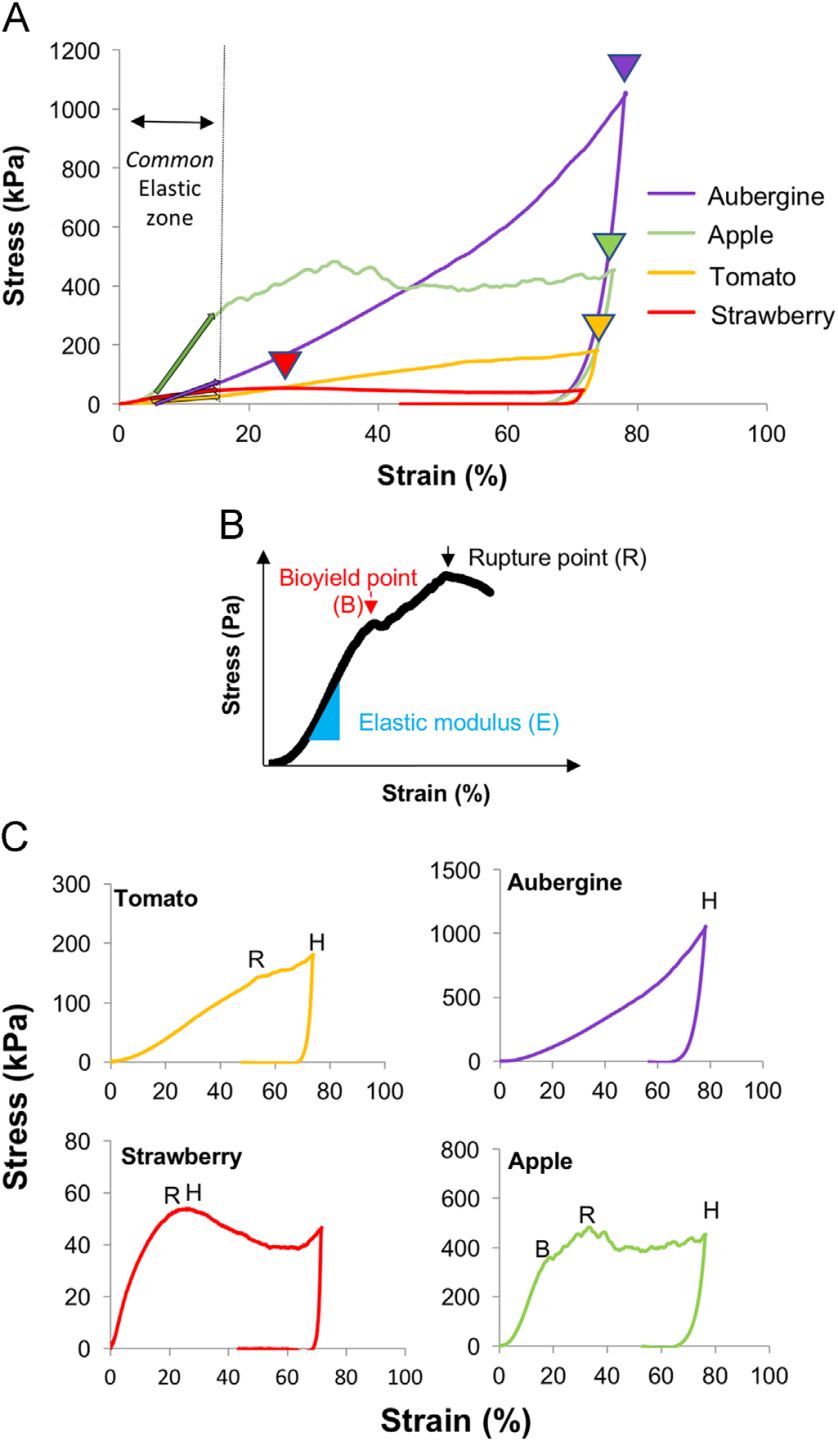
Stress-strain curves from texture analysis by compression test at maximum strain of 70%. A) Typical stress-strain curves of the four fruits to represent the elastic modulus (*E*) defined by the slope in the elastic zone of these four fruits, and depicted by a straight and thicker line, and the hardness (*H*) by an arrowhead, both of its respective color. B) A representative stress-strain diagram from an agricultural product including three biologically meaningful texture parameters: elastic modulus (*E*), bioyield point (*B*) and rupture point (*R*). C) Zoom of each individual stress-strain curve to include bioyield (*B*) and rupture (*R*) points when present, in contrast to hardness (*H*), each marked by the corresponding initial letter.

Fig. 1A includes a typical stress-strain curve for each fruit species. The aubergine profile has its only peak force at maximum strain, which is considered as hardness (*H*) (Fig. 1C), drawing a curve quasi exponential without any bioyield or rupture points (see Fig. 1B for clarification of bioyield and rupture points). In contrast, when firmness is determined by the elastic modulus (*E*) (Fig. 1A), apple is the stiffer fruit, with the steepest slope of all fruits, followed by the bioyield and ruptures points, after which a jagged profile depicts the multiple tissue fractures of the typical apple crispness (Fig. 1C). Finally, tomato and strawberry fruits showed both the lowest values of elastic modulus (*E*) and hardness (*H*), producing curves with deflection points, instead of acute peaks like in aubergine or apple, that reflects the characteristic soft and melting texture of these two fleshy fruits (Fig. 1C). Fig. 1B includes the three texture parameters that are scientifically rigorous and possess biological meaning: the elastic modulus (*E*) as a measurement of the stiffness of the sample; the bioyield point (*B*) that appears in some fruits and corresponds to an initial-local rupture of the sample at cellular level; and rupture point (*R*) that corresponds to a massive failure at tissue level.

### 1.2 Epitope mapping of sequentially extracted cell wall polysaccharides in the four fruits

**Fig. 2:**
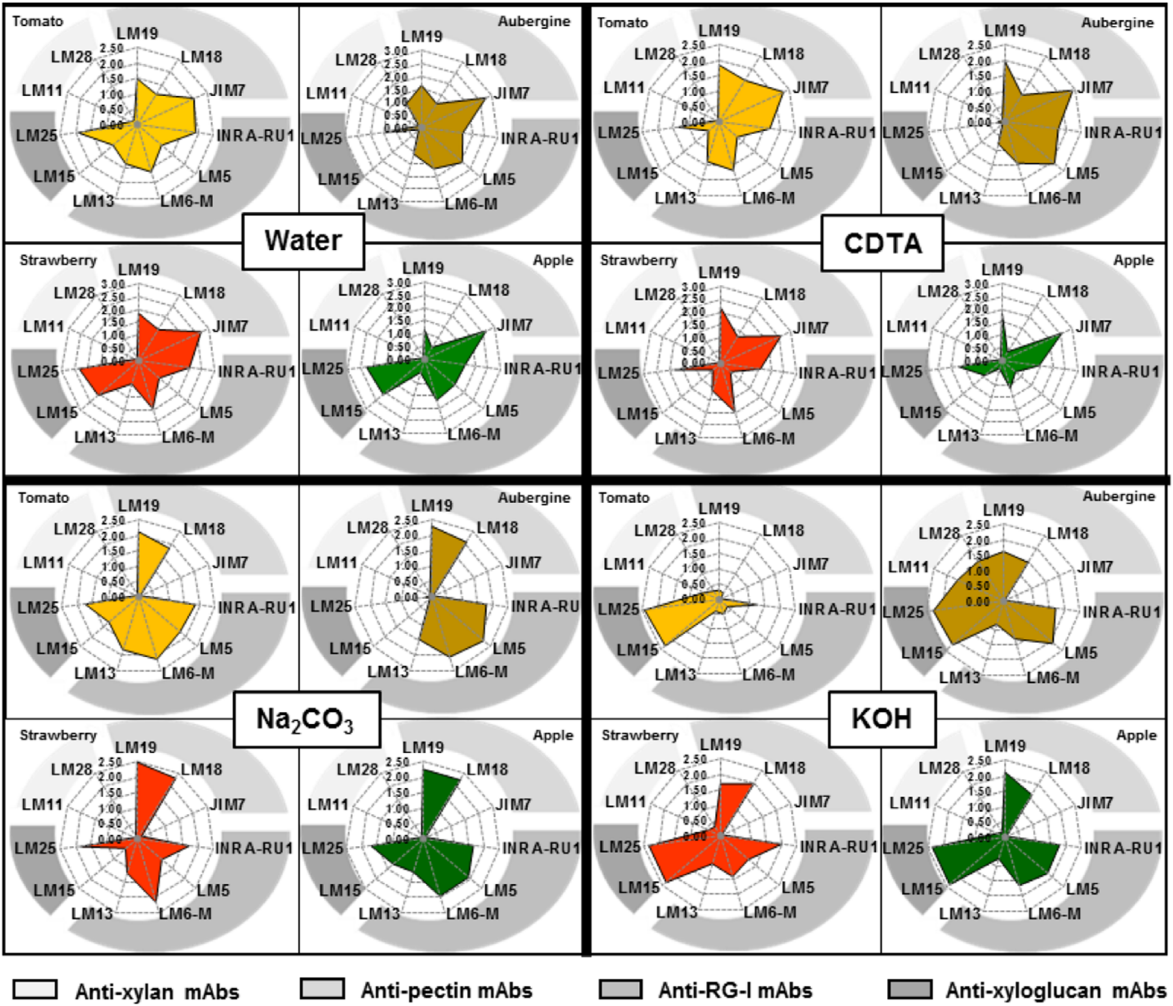
Overview of cell wall matrix polysaccharide epitope levels grouped as pectic HG, pectic RG-I, xyloglucan and heteroxylan in water, CDTA, Na_2_CO_3_, and KOH fractions from the four fruits.

See Fig. 2 for the immunochemical analysis performed on the four fruit parenchyma systems extracted sequentially using water, CDTA, 1 M Na_2_CO_3_ and 4 M KOH. Methylesterified homogalacturonan (HG) (JIM7) was the most strongly detected component in water and CDTA extracts in all four fruits. Unesterified HG (LM19) and rhamnogalacturonan-I (RG-I) (INRA-RU1) were detected in all fractions. RG-I side chain epitopes were differently distributed with galactan (LM5) being predominant in aubergine and arabinan (LM6-M) in tomato and strawberry. Xyloglucan was detected in all four species in water and KOH fractions. The LM15 xyloglucan epitope was abundantly detected in apple and strawberry water extracts but not in tomato or aubergine. Xylan epitopes (LM11 and LM28) were only found in the water and KOH extracts of aubergine.

## 2 Experimental design, materials and methods

Tomato (*Solanum lycopersicum* L.), apple (*Malus domestica* Borkh. cv. Granny Smith) and aubergine (*Solanum melongena* L.) were purchased locally and processed at the point of consumer use. Strawberries (*Fragaria x ananassa* Duchesne) were grown with a 16:8 h light:dark cycle at 25 °C and harvested when ripe.

Identical cylindrical pieces of parenchyma (5 mm × 9 mm) obtained with a cork borer from the ripe outer pericarp (not including skin), were selected for mechanical assays. This simplification was used for comparative purposes as they are homogeneous tissues and the main edible portion of the fruit [2]. Texture parameters were measured with a texturometer (Texture analyser TA-XT 2i: *Ametek, Lloyd Instruments Ltd., Fareham, UK*) through the uniaxial compression test that measures the force required to compress the fruit cylinder between two steel plates (comparable with a whole-hand squeeze). The compression assay parameters were: compression plate P75; speed test 1 mm/s; target mode strain; strain 70%; and trigger force 5 g. From the resulting stress-strain curves two parameters were analyzed: hardness (*H*) defined by the maximum force, which is widely used in food industry; and the elastic modulus (*E*), defined by the slope of the curve in the elastic zone of the sample (where the initial shape is recovered when the stress ceased), and considered a scientifically rigorous measurement of stiffness. Texture assays were done in triplicate. Soluble solids were measured using a refractometer MT-032ATC (TR Turoni, Italy) and were expressed as % Brix. Percentage of moisture content was estimated by the weight loss of fresh material after complete drying in an oven at 80 °C for 24 h.

Cell wall extracts from the four fruit parenchyma systems were prepared as detailed in [1]. ELISA screening of cell wall epitope occurrence in all fruits fractions were performed by coating each extract (water, CDTA, Na_2_CO_3_ and KOH) onto microtitre plates using 100 µl of the extracts diluted 60-fold in 1× PBS (phosphate-buffered saline: 137 mM NaCl, 2.7 mM KCl, 10 mM Na_2_HPO_4_, 2 mM KH_2_PO_4_) overnight at 4 °C. The ELISA analysis was performed following the protocol described in [3]. The analysis of each sample was performed in triplicate. Due to varied antibody avidities, direct quantitative comparisons between antibodies are limited but the data can be used for quantitative comparison of epitope levels between fruits and extractions.

## Funding sources

This research was supported by a Marie Curie Intra European Fellowship to SP within the 7th European Community Framework Programme (PIEF-GA-2013-625270). The work was also supported by the United Kingdom Biotechnology and Biological Research Council (BBSRC, grant BB/K017489/1).
