# Supplementary material for: Extraction, texture analysis and polysaccharide epitope mapping data of sequential extracts of strawberry, apple, tomato and aubergine fruit parenchyma
Source: Data Brief. 2018 Jan 31;17:314–20. doi: 10.1016/j.dib.2018.01.013 (PMC5988314; doi:10.1016/j.dib.2018.01.013)
Supplement: Supplementary file 1 — Supplementary material [file mmc1.docx]

Data in Brief manuscript entitled: **Extraction, texture analysis and polysaccharide epitope mapping data of sequential extracts of strawberry, apple, tomato and aubergine fruit parenchyma**. Ref: DIB-D-17-01136R1

**Declaration of conflicts of interest**: none
